# Heterogeneous effects of increased availability of alcohol on hospitalization due to external causes: quasi-experimental evidence from the introduction of Saturday opening at Swedish alcohol retail stores

**DOI:** 10.1093/aje/kwae208

**Published:** 2024-07-16

**Authors:** Ylva B Almquist, Lars Brännström, Anders Hjorth-Trolle, Mikael Rostila

**Affiliations:** Department of Public Health Sciences, Centre for Health Equity Studies (CHESS), Stockholm University, Stockholm, Sweden; Department of Social Work, Stockholm University, Stockholm, Sweden; The ROCKWOOL Foundation, Copenhagen, Denmark; Department of Public Health Sciences, Centre for Health Equity Studies (CHESS), Stockholm University, Stockholm, Sweden

**Keywords:** policy reforms, alcohol consumption, external causes, experiment, Sweden

## Abstract

Responses to increased alcohol availability may vary across the population as a function of differential vulnerability. This study therefore aimed to examine the effects of the implementation of Saturday opening at the Swedish alcohol retail monopoly in 2000 on risks of hospitalization due to external causes (HEC) among different population subgroups. Leveraging the experimental design of the reform, longitudinal difference-in-differences analyses were applied to a register-based cohort of individuals aged 20-40 at the time of implementation. The population was stratified into groups of Swedish, Finnish, and Middle Eastern origin, known to represent different levels of alcohol consumption and rates of alcohol-related morbidity. Results showed a 17.7% increase (*P < .*029) in the risk of HEC among individuals of Finnish origin, as jointly caused by both increased prevalence in the experiment area and decreased prevalence in the control area. The increase was primarily driven by younger men with lower levels of education. Those of Swedish origin exhibited largely similar patterns (9.7% increase; *P < .*001), while no measurable impact was observed among individuals of Middle Eastern origin (−21.4% decrease; *P < .*076). The findings confirm that increasing alcohol availability contributes to the disease burden related to alcohol among population subgroups already susceptible to its effects.

## Introduction

Previous research has confirmed causal links between alcohol consumption and poor health, as indicated by a wide range of diseases and injury conditions, which explains why a considerable part of the global burden of disease can be attributed to alcohol.^1^^,^^2^ In attempting to reduce the adverse health-related consequences of alcohol consumption at the population level, a common approach has been to target the availability of alcohol.^3^ Public health policies focused on reducing availability—for example, state regulations regarding minimum legal drinking age, the establishment of alcohol monopolies, and restrictions on the type, number, and opening hours of retail stores—generally seem to be efficient means to reduce alcohol consumption^4^ and, in turn, alcohol-related health consequences.^5^^‑^^7^ By contrast, increasing availability tends to result in elevated levels of alcohol consumption.^4^ Comparatively less is known, however, about whether the effect of increased availability extends beyond consumption to further manifest itself through various health-related consequences. Existing studies have provided mixed results,^8^^,^^9^ implying a potential asymmetry in effects of alcohol policy reforms. On the one hand, this suggests that those who increase their consumption in light of increasing alcohol availability may not necessarily be the same individuals who consume less alcohol when availability is reduced. On the other hand, the null findings may obscure significant variations in responses to increased alcohol availability, emphasizing the importance of examining how these effects may differ across population groups.

The Swedish context might offer a particularly interesting case for exploring health outcomes following increases in alcohol availability. Sweden has long had a restrictive alcohol policy with strict regulations regarding availability and marketing, as well as high taxes on alcohol. Sales are largely limited to the state-owned alcohol retail monopoly, “Systembolaget.”^10^ In the 2000s, Systembolaget’s sales hours were expanded from weekdays only to also include Saturdays. This reform was implemented in 2 phases. The first phase was initiated in February 2000 and affected only part of Sweden. In the second phase, starting in July 2001, Saturday opening was extended to the whole country. The increase in consumption after the reform has been estimated to approximately 4%.^11^^‑^^13^ The same studies have additionally looked into consequences of this elevated consumption, but the results do not provide any consistent picture regarding effects on outcomes such as drink-driving and other types of crime (violent crimes, such as assault and property crimes). From a public health perspective, there are nonetheless several good reasons to revisit the impact of the reform. Perhaps the most important one is that crime rates do not reflect the fuller scope of health-related outcomes that could follow directly from harmful patterns of alcohol consumption. Here, hospitalization due to external causes (hereafter HEC)—which includes accidents, intentional self-harm, assault, and events of undetermined intent—might offer a more public health–relevant estimation of the effects of the reform.^1^

Drawing on longitudinal population-based register data, this study thus aims to assess the impact of the Saturday opening reform on the risk of HEC. Underlying reasons for HEC are differently distributed across age: in younger age groups, HEC is primarily driven by behavior (eg, traffic accidents, self-harm, and violence) whereas in older age groups, reasons more strongly reflect fall injuries and complications related to health care. Therefore, we restrict our study to individuals aged 20-40 at the time of the reform. We will compare individuals living in the 6 regions (experiment area) affected in the first phase with individuals living in 7 unaffected regions (control area). Importantly, we will contrast the effect of the reform across population subgroups. These subgroups have been selected to reflect known differences in vulnerability to alcohol.^9^^,^^14^^,^^15^ To begin with, we will compare 3 population subgroups defined by ethnic background: Swedish-born individuals who have Swedish-born parents, and individuals of Finnish and Middle Eastern origins, respectively. Alcohol per capita consumption is higher in Finland than in Sweden, ^16^ whereas consumption levels are much lower across the Middle East, with alcohol even being prohibited in some countries.^17^ These differences are echoed in the patterns of alcohol-related morbidity across the corresponding population groups in Sweden.^18^ Furthermore, within each of these 3 subgroups, differences in the impact of the reform by sex, birth cohort, and educational level will be explored. The rationale is that previous research has shown men and individuals in younger age groups to both consume more alcohol and be more adversely affected by their consumption,^19^ whereas lower-educated individuals display higher rates of alcohol-related harms despite the fact that they tend to drink comparatively less than higher-educated groups.^20^ Finally, compared to first-generation migrants, rates among second-generation migrants tend to be more similar to those of the native population.^21^ Hence, we will stratify the Finnish and Middle Eastern populations based on generation.

## Methods

### The experiment

In preparation of the reform, all regions (then called counties) in Sweden (except for Gotland) were divided into 3 areas: experiment area (Norrbotten, Västerbotten, Jämtland, Västernorrland, Stockholm, Skåne), buffer area (Dalarna, Gävleborg, Uppsala, Södermanland, Halland, Kronoberg, Blekinge), and control area (Värmland, Örebro, Västmanland, Östergötland, Kalmar, Jönköping, Västra Götaland). The areas were chosen to represent a variation in the degree of urbanization and geographical location and to have approximately the same population size. The purpose of the buffer area was to decrease the risk of spill-over effects into the control area. Details concerning the implementation have been published elsewhere.^12^^,^^13^

### Study sample

We used Swedish longitudinal register data to identify individuals who lived in either the experiment area or the control area between 1998 and 2001 (individuals living in the buffer area, or moving between areas, were excluded), who were born from 1959-1979, and remained alive during the follow-up of HEC (*n* = 1 782 412).

In this study, the total population represents only a point of reference. Our main focus is placed on the 3 subgroups formed on the basis of ethnic background: “Swedish” (Swedish-born with Swedish-born parents; *n* = 1 189 718), “Finnish” (Finnish-born or Swedish-born with at least 1 Finnish-born parent; *n* = 113 038), and “Middle Eastern” (Middle Eastern-born or Swedish-born with at least 1 Middle Eastern-born parent; *n* = 45 848). The information used to construct these samples were derived from Statistics Sweden’s database for integration studies (STATIV) combined with the multigenerational register.

Ethical permission for the research performed in this study was granted by the Swedish Ethical Review Authority (decision no. 2021-06797-01).

#### Outcome variable

Our outcome was HEC, as recorded in the National Patient Register, from November 1, 1998, until April 30th, 2001. This register is kept by the Swedish National Board of Health and Welfare and contains all inpatient care events with at least 1 overnight stay in a Swedish hospital. We considered all records including any of the diagnoses found in Chapter XX (diagnoses V01-Y98, based on the 10th revision of the International Classification of Diseases; ICD 10), which primarily reflect accidents, intentional self-harm, assault, and events of undetermined intent (thus including accidental and nonaccidental poisoning by alcohol). Due to data limitations, we cannot discern all subchapters of Chapter XX. The distribution of individuals across the subchapters that are possible to identify, is shown in Table S1. As can be noted, some groups are too heterogeneous, or contain too few individuals, to motivate a subchapter analysis.

We divided the follow-up of HEC into 10 time intervals that spanned from November 1998 to January 2001, each covering 3 months. The choice of 3-month intervals was due to HEC being a comparably rare group of diagnoses and the need to maintain a sufficiently high prevalence of hospitalized individuals within each interval. Limiting the number of time intervals to 10 meant that our analysis covered 15 months before and after the reform, respectively. Shorter pre-intervention and postintervention periods would decrease the number of time intervals in the model and potentially produce less reliable results. Longer periods, on the other hand, could risk capturing other changes occurring at the macro level. In particular, we wanted to avoid reaching too close to the second implementation phase of the reform.

#### Stratification variables

The following set of stratification variables were included: sex, birth cohort, educational level, and generation. For more details on data sources and categorization, see Table 1.

**Table 1:** Overview of stratification variables.

**Variable**	**Categorization**	**Additional information**
Sex	(0) Men (1) Women	
Birth cohort	(1) Born 1959-1965 (2) Born 1966-1972 (3) Born 1973-1979	The upper bound was chosen to ensure that all individuals had turned 20 years of age in February 2000, and thus had reached the legal age for buying alcohol at Systembolaget. As alluded to in the introduction, the choice of lower bound was made due to risk consumption of alcohol being higher in younger as compared to older age groups.^19^ Moreover, with age, rates of behaviorally caused HEC decrease whereas rates of nonbehaviorally caused HEC increase. Another important reason is that the coverage of information about country of origin becomes poorer the lower the birth year.
Educational level	(1) Compulsory education (2) Upper sec. education (3) University education (4) Missing education	Information on educational level was retrieved from Statistics Sweden’s Longitudinal Integration Database for Health Insurance and Labour Market Studies (LISA) and refers to the year 2000.
Generation	(0) First generation (1) Second generation	First-generation migrants include individuals who were born in Finland and the Middle East, respectively. Second-generation migrants include those who were born in Sweden but had at least 1 parent born in Finland or the Middle East, respectively.

### Statistical analysis

To estimate the impact of Saturday opening on HEC, we applied a longitudinal difference-in-differences (DID) approach.^22^ This approach enabled an assessment of the change in outcome between the pre-experiment period and postexperiment period among the individuals living in the experiment area and compared it to the corresponding change among individuals living in the control area. In doing so, we were able to control for unobserved time-invariant individual-level characteristics that might be correlated with the reform and the outcome. Our DID analysis was specified as a linear regression model for panel data and estimated by means of the *xtdidregress* command in Stata 17/SE.^23^ Since the outcome is binary, the estimated model becomes a linear probability model. The reported DID estimates thus express absolute differences in probabilities (ie, risk differences). In practice, we estimate the following model:


\begin{align*} {HEC}_{i,t}&={\beta}_0+{\beta}_1{AREA}_i+{\beta}_2{POSTREFORM}_{i,t}+{\beta}_3{AREA}_i\\&\quad\ast{POSTREFORM}_{i,t}+{e}_{i,t} \end{align*}

${AREA}_i$ is a dummy measuring if an individual lives in the experiment area and which does not vary over time (since we exclude movers), ${POSTREFORM}_{i,t}$ is a dummy indicating if the individual was measured before or after the reform was introduced, and ${e}_{i,t}$ is an error term. Standard errors are clustered by area to account for correlated errors within area.

#### Assumptions

The change among individuals living in the control area is assumed to be an estimate of what would have happened among individuals living in the experiment area if there had been no reform (ie, the true counterfactual). While we cannot directly test this assumption, we assess whether the time trends during the pre-experiment period were the same in the experiment area as in the control area. If the trends have been the same (ie, parallel), then it is probable that they would have been the same in the postexperiment period if the individuals living in the experiment area had not been exposed to the reform.

Figure S1 plots the observed mean probabilities of HEC on the left-hand side of each panel. Fluctuation can be noted here due to the low prevalence of HEC. The right-hand side of each panel is based on an augmented DID model specified to capture differences in slopes between the control area and experiment area across the pre-experiment period. The model centers the time variable around its minimum value, making it easier to detect deviations from parallelism. These linear trend plots indicate that the trends are parallel for the total, Swedish, Finnish, and Middle Eastern samples. This is also confirmed by the results from the Wald tests presented in Table S2. In general, there is not sufficient evidence to reject the assumption of parallel trends in any of the subgroup analyses either. Additionally, we report the results from a Granger-type of causality test, which assesses whether the hypothesized effect of the reform is observed prior to the change (Table S2; Figure S2). The results here do generally not provide any evidence of anticipatory effects.

Under this assumption of parallel trends, the difference between the change in the experiment area and the control area are then estimates of the causal effect of the reform on HEC. Note that this model also assumes exogeneity with respect to factors that (1) vary over time, (2) affect HEC, (3) differ between the experiment and control area, and/or affect HEC differently in the experiment area and control area, and (4) did not occur outside the exact reform time window (since this would then be caught by the tests for parallel trends). While we cannot explicitly test for bias from factors that meet all 4 conditions, we note that our time window is very short, and our parallel trends hold. Thus, we deem this assumption plausible.

### Additional analyses

We conducted 4 types of additional analysis to ensure the validity of our findings.

First, although we initially observed a reasonable balance between the experiment and control groups in terms of the included covariates (Table S3), we performed coarsened exact matching (CEM) to address any potential imbalance (Table S4). This technique is akin to synthetic control analysis but better suited for our longitudinal difference-in-differences (DID) design. Subsequently, we re-estimated the main results, incorporating weights derived from the CEM analysis (Table S5).

Second, to further strengthen our analysis, we re-estimated the main results using cancer as the outcome instead of HEC (Table S6). This approach served as a placebo check, as the reform should not have any discernible effects on this outcome.

Third, in the main analysis, the pre-intervention period covered 15 months. While this already extends back in time quite significantly, we acknowledge that our assessment of the parallel trends assumption relies on only 5 measurement intervals, which might increase the risk of a type II error. Therefore, we extended the pre-intervention period to 10 3-month intervals (Table S7).

Fourth, we analyzed the impact of the reform across both implementation waves. Using staggered or stacked DID models is a common statistical approach to analyzing policies rolled out in consecutive steps. It has nonetheless been heavily criticized in recent years for introducing severe biases and relying on unrealistic assumptions, hence resulting in uncredible effect estimations.^24^^‑^^27^ We therefore provide the results only as a reference and without using it to draw any further conclusions about our main results. More details on the setup of the model and its inherent problems are presented in the supplements (Table S8).

## Results

### Descriptive statistics

According to the descriptives in Table 2, a majority of the total sample lived in the experiment area during the study period, the proportions of men and women are around the same, a lower proportion of individuals belongs to the youngest birth cohort, and most individuals had obtained upper secondary education. It was somewhat more common among those of Finnish and Middle Eastern origins to live in the experiment area. These samples were generally also older and had lower education compared to the Swedish sample. Moreover, the Middle Eastern sample consisted of more men in comparison to those of Swedish and Finnish origins. With regards to generation, around 3-quarters of Finnish individuals were second-generation migrants, whereas this is only the case for a very small portion of the Middle Eastern sample.

**Table 2:** Descriptive statistics for the study variables, stratified by ethnic background.

	**Total** ^**a**^ **(*N* = 1 782 412)**				**Sweden** ^**b**^ **(N = 1 189 718)**				**Finland** ^**c**^ **(*N* = 113 038)**				**Middle East** ^**d**^ **(*N* = 45 848)**			
	**Control area (*N* = 789 274)**		**Experiment area (*N* = 993 138)**		**Control area (*N* = 548 831)**		**Experiment area (*N* = 640 887)**		**Control area (*N* = 46 341)**		**Experiment area (*N* = 66 697)**		**Control area (*N* = 18 716)**		**Experiment area (*N* = 27 132)**	
	*n*	%	*n*	%	*n*	%	*n*	%	*n*	%	*n*	%	*n*	%	*n*	%
Sex																
Men	406 984	51.6	502 379	50.6	285 120	52.0	326 846	52.0	23 826	51.4	32 518	48.8	10 199	54.5	14 475	53.4
Women	382 290	48.4	490 759	49.4	263 711	48.0	314 041	48.0	22 515	48.6	34 179	51.2	8517	45.5	12 657	46.7
Birth cohort																
Born 1959-1965	297 552	37.7	370 978	37.4	188 368	34.3	217 298	33.9	18 684	40.3	26 623	39.9	8559	45.1	12 131	44.7
Born 1966-1972	287 987	36.5	367 400	37.0	205 860	37.5	244 190	38.1	17 367	37.5	24 797	37.2	6204	33.2	9170	33.8
Born 1973-1979	203 735	25.8	254 760	25.7	154 603	28.2	179 399	28.0	10 290	22.2	15 277	22.9	4063	21.7	5831	21.5
Educational level																
Compulsory education	99 820	12.7	115 827	11.7	57 679	10.5	58 209	9.1	7249	15.6	9113	13.7	4836	25.8	6522	24.0
Upper sec. Education	457 333	57.9	515 756	51.9	322 686	58.8	339 427	53.0	28 881	62.3	37 670	56.5	8188	43.8	11 107	40.9
University education	226 826	28.7	350 716	35.3	167 094	30.5	241 750	37.7	10 031	21.7	19 325	29.0	4936	26.4	8384	30.9
Missing education	5295	0.7	10 839	1.1	1372	0.3	1501	0.2	180	0.4	589	0.9	756	4.0	1119	4.1
Generation																
First generation	94 034	39.1	154 643	43.9					11 254	24.3	18 101	27.1	18 499	98.8	26 400	97.3
Second generation	146 409	60.9	197 608	56.1					35 087	75.7	48 596	72.9	217	1.2	732	2.7

^a^The whole population regardless of ethnic background.

^b^Swedish-born with Swedish-born parents.

^c^Finnish-born or Swedish-born with a Finnish-born parent.

^d^Middle Eastern-born or Swedish-born with a Middle Eastern-born parent.

Table 3 shows the prevalence of HEC across the study variables. The prevalence in the total sample is nearly 2%. A statistically significantly higher prevalence is noted among those of Finnish origin, men, younger birth cohort, individuals with lower educational level, and second-generation migrants.

**Table 3:** Differences in frequency and prevalence of HEC (November 1998—April 2001), stratified by ethnic background.

	**Total** ^**a**^ **(N = 1 782 412)**				**Sweden** ^**b**^ **(N = 1 189 718)**				**Finland** ^**c**^ **(*N* = 113 038)**				**Middle East** ^**d**^ **(*N* = 45 848)**			
	**Control area (*N* = 789 274)**		**Experiment area (*N* = 993 138)**		**Control area (*N* = 548 831)**		**Experiment area (*N* = 640 887)**		**Control area (*N* = 46 341)**		**Experiment area (*N* = 66 697)**		**Control area (*N* = 18 716)**		**Experiment area (*N* = 27 132)**	
	*n*	%	*n*	%	*n*	%	*n*	%	*n*	%	*n*	%	*n*	%	*n*	%
Overall	15 654	1.98	19 097	1.92	10 400	1.89	11 948	1.86	1510	2.26	1177	2.54	574	2.12	399	2.13
Sex																
Men	9723	2.39	11 597	2.31	6621	2.32	7326	2.24	875	2.69	708	2.97	345	2.38	228	2.24
Women	5931	1.55	7500	1.53	3779	1.43	4622	1.47	635	1.86	469	2.08	229	1.81	171	2.01
Birth cohort																
Born 1959-1965	5642	1.90	6868	1.85	3834	1.76	3316	1.76	592	2.22	488	2.61	241	1.99	147	1.74
Born 1966-1972	5295	1.84	6625	1.80	4182	1.71	3605	1.75	559	2.25	403	2.32	189	2.06	140	2.26
Born 1973-1979	4717	2.32	5604	2.20	3932	2.19	3479	2.25	359	2.35	286	2.78	144	2.47	112	2.76
Educational level																
Compulsory education	3149	3.15	3722	3.21	1899	3.26	1829	3.17	343	3.76	286	3.95	166	2.55	126	2.61
Upper sec. Education	9505	2.08	10 832	2.10	6977	2.06	6506	2.02	901	2.39	721	2.50	257	2.31	165	2.02
University education	2856	1.26	4354	1.24	3026	1.25	2006	1.20	242	1.25	164	1.63	125	1.49	86	1.74
Missing education	144	2.72	189	1.74	46	3.06	59	4.30	24	4.07	6	3.33	26	2.32	22	2.91
Generation																
First generation	1999	2.13	2950	1.91					435	2.40	333	2.96	563	2.13	396	2.14
Second generation	3255	2.22	4199	2.12					1075	2.21	844	2.41	11	1.50	3	1.38

Abbreviation: HEC, Hospitalization due to external causes.

^a^The whole population regardless of ethnic background.

^b^Swedish-born with Swedish-born parents.

^c^Finnish-born or Swedish-born with a Finnish-born parent.

^d^Middle Eastern-born or Swedish-born with a Middle Eastern-born parent.

In Table 4, the prevalence of HEC across the time intervals is demonstrated, divided into the experiment area and control area, and stratified by ethnic background.

**Table 4:** Descriptives of HEC across 3-month intervals (November 1998—April 2001), stratified by ethnic background.

		**Hospitalization due to external causes**							
		**Total** ^a^ (*n* = 1 782 412)		**Sweden** ^b^ (n = 1 189 718)		**Finland** ^c^ (*n* = 113 038)		**Middle East** ^d^ (*n* = 45 848)	
		*n*	%	*n*	%	*n*	%	*n*	%
Pre-intervention period	November 1998 to January 1999								
	Control area	1646	0.209	1113	0.203	135	0.291	40	0.214
	Experiment area	2003	0.202	1265	0.197	159	0.238	58	0.214
	February 1999 - April 1999								
	Control area	1770	0.224	1153	0.210	138	0.298	42	0.224
	Experiment area	2037	0.205	1249	0.195	163	0.244	75	0.276
	May 1999 - July 1999								
	Control area	1938	0.246	1300	0.237	127	0.274	33	0.176
	Experiment area	2395	0.242	1519	0.237	194	0.291	71	0.262
	August 1999 - October 1999								
	Control area	1938	0.246	1276	0.233	161	0.347	49	0.262
	Experiment area	2067	0.208	1297	0.202	160	0.240	56	0.206
	November 1999 - January 2000								
	Control area	1671	0.212	1142	0.208	124	0.268	43	0.230
	Experiment area	2107	0.212	1287	0.201	157	0.235	72	0.265
Post-intervention period	February 2000 - April 2000								
	Control area	1574	0.199	1041	0.190	107	0.231	42	0.224
	Experiment area	2215	0.214	1328	0.207	169	0.253	64	0.236
	May 2000 - July 2000								
	Control area	1756	0.223	1168	0.213	122	0.263	52	0.278
	Experiment area	2160	0.218	1392	0.217	178	0.267	54	0.199
	August 2000 - October 2000								
	Control area	1765	0.224	1166	0.213	134	0.289	43	0.230
	Experiment area	2145	0.216	1350	0.211	191	0.286	49	0.181
	November 2000 - January 2001								
	Control area	1642	0.208	1064	0.194	146	0.315	50	0.267
	Experiment area	2207	0.222	1360	0.212	198	0.297	60	0.221
	February 2001 - April 2001								
	Control area	1671	0.212	1096	0.200	114	0.246	42	0.224
	Experiment area	2088	0.210	1312	0.205	154	0.231	67	0.247

Abbreviation: HEC, Hospitalization due to external causes.

^a^The whole population regardless of ethnic background.

^b^Swedish-born with Swedish-born parents.

^c^Finnish-born or Swedish-born with a Finnish-born parent.

^d^Middle Eastern-born or Swedish-born with a Middle Eastern-born parent.

### Effects of the reform on HEC

Table 5 reports the absolute differences in probabilities of HEC. There is an adverse effect of the reform (0.00016, *P < .*001) in the total population, corresponding to a 7.5% increase of HEC. A similar-sized estimate is presented for the Swedish sample (0.00020, *P < .*001; 9.7 % increase), whereas it is larger among those of Finnish origin (0.00044, *P* = 0.029; 17.7% increase). The estimate for the Middle Eastern sample is negative but statistically nonsignificant (−0.00052, *P* = 0.076; 21.4% decrease).

**Table 5:** Effects of Saturday opening at Systembolaget on the probability of HEC across 3-month intervals (November 1998—April 2001). Results from fixed-effects DID models.

	**Estimate** ^**a**^	**Robust Std. Err.**	***P* value**	**Comparison to baseline** ^**b**^
Total (*n* = 1 782 412)	**0.00016**	0.00005	**<0.001**	**7.5 %**
Stratified by sex				
Men	**0.00025**	0.00025	**<0.001**	
Women	0.00007	0.00006	0.216	
Stratified by birth cohort				
Born 1959-1965	0.00018	0.00007	0.134	
Born 1966-1972	**0.00018**	0.00072	**0.013**	
Born 1973-1979	**0.00023**	0.00010	**0.019**	
Stratified by educational level				
Compulsory education	−0.00004	0.00017	0.817	
Upper sec. Education	**0.00021**	0.00006	**0.001**	
University education	**0.00015**	0.00006	**0.015**	
Missing education	0.00030	0.00056	0.589	
Stratified by generation				
First generation	−0.00016	0.00012	0.190	
Second generation	**0.00025**	0.00011	**0.021**	
Sweden (*n* = 1 189 718)	**0.00020**	0.00005	**<0.001**	**9.7 %**
Stratified by sex				
Men	**0.00028**	0.00008	**0.001**	
Women	0.00011	0.00007	0.099	
Stratified by birth cohort				
Born 1959-1965	0.00015	0.00009	0.098	
Born 1966-1972	**0.00017**	0.00008	**0.044**	
Born 1973-1979	**0.00031**	0.00011	**0.005**	
Stratified by educational level				
Compulsory education	0.00018	0.00023	0.438	
Upper sec. Education	**0.00023**	0.00008	**0.002**	
University education	**0.00016**	0.00007	**0.033**	
Missing education	0.00006	0.00153	0.967	
Finland (*n* = 113 038)	**0.00044**	0.00020	0.029	**17.7 %**
Stratified by sex				
Men	**0.00065**	0.00031	**0.033**	
Women	0.00022	0.00026	0.387	
Stratified by birth cohort				
Born 1959-1965	0.00026	0.00032	0.414	
Born 1966-1972	0.00038	0.00031	0.234	
Born 1973-1979	**0.00085**	0.00043	**0.049**	
Stratified by educational level				
Compulsory education	0.00048	0.00066	0.467	
Upper sec. Education	**0.00053**	0.00026	**0.042**	
University education	0.00028	0.00031	0.375	
Missing education	−0.00068	0.00420	0.872	
Stratified by generation				
First generation	0.00009	0.00041	0.836	
Second generation	**0.00057**	0.00023	**0.014**	
Middle East (n = 45 848)	−0.00052	0.00029	0.076	-21.4
Stratified by sex				
Men	−0.00074	0.00041	0.069	
Women	−0.00026	0.00041	0.527	
Stratified by birth cohort				
Born 1959-1965	0.00002	0.00039	0.958	
Born 1966-1972	−0.00067	0.00051	0.191	
Born 1973-1979	−0.00138	0.00072	0.054	
Stratified by educational level				
Compulsory education	−0.00103	0.00064	0.105	
Upper sec. Education	0.00013	0.00045	0.772	
University education	**−0.00118**	0.00047	**0.012**	
Missing education	−0.00009	0.00162	0.958	
Stratified by generation				
First generation	−0.00053	0.00029	0.069	
Second generation	0.00027	0.00244	0.911	

Abbreviations: HEC, Hospitalization due to external causes; DID, Difference-in-differences.

Estimates in bold signifies *P < .*05.

^a^Estimates are absolute differences in probabilities (risk differences).

^b^“Comparison to baseline” shows the estimate presented in percentage change in risk of HEC.

Overall, the estimates are notably larger among men in the total population as well as the Swedish and Finnish samples, whereas no statistically significant effect on HEC among women is observed in any of the samples. Furthermore, there is a tendency for the effect to be more pronounced among the youngest birth cohort, particularly among those of Finnish origin. No discernible gradient is observed based on educational level. Instead, the probability of HEC seems to be highest among individuals with upper secondary education. A statistically significant effect of the reform is demonstrated for second-generation migrants in the total population and Finnish sample but not for first-generation migrants in any of the samples. It can be noted that none of the subgroup analyses for the Middle Eastern sample yield statistically significant results, except for individuals with a university education, where a relatively large negative estimate is observed.

### Results from the additional analyses

After incorporating weights derived from coarsened exact matching (CEM) analysis in our fixed-effects difference-in-differences (DID) models (Table S5), we conclude that the main findings remain consistent. Moreover, our analysis indicates no discernible impact of the reform on hospitalization due to cancer (Table S6), aligning with our initial expectations. The results based on the extended pre-intervention period (Table S7) show that the assumption of parallel trends holds for the Finnish and Middle Eastern samples but is violated for the total and Swedish samples. The overall effects among these latter 2 samples should therefore be approached with caution. Lastly, when performing the analysis jointly across the 2 implementation phases of the reform, smaller effect sizes were observed, but the general pattern was intact (Table S8).

## Discussion

The aim of this study was to investigate the impact of the introduction of Saturday opening at the Swedish retail monopoly Systembolaget in February 2000 on HEC among individuals aged 20-40 at the time of the reform, using a longitudinal DID approach. We were particularly interested in exploring potential heterogeneity in responses to the reform across population subgroups known to have different vulnerabilities to alcohol consumption and its associated health consequences. To achieve this, we stratified the population into individuals of Swedish, Finnish, and Middle Eastern origin, and further analyzed these subgroups based on sex, birth cohort, educational level, and generation.

While we are unaware of any previous research examining the broader public health consequences of the Saturday opening reform, a recent study^28^ might offer an interesting reference point. That study explored the effects of extended opening hours of Swedish alcohol retail stores on various health outcomes, including alcohol-related hospital admissions, mortality, traffic accidents, health-related work absence, mental health, and self-reported health. The results did not demonstrate any notable health impact, despite the evidence indicating increased alcohol consumption. This aligns with several earlier studies on changes in alcohol availability, contributing to the idea of an asymmetry in effects. By contrast, our findings do generally not support the notion of asymmetry. We observed a statistically significant effect across the period November 1998 to April 2001, reflecting a 7.5% increase of HEC in the total population. This estimate is somewhat larger than expected, provided that previous research has shown a consumption increase of 4% and an elasticity of around 0.5.^11^^‑^^13^ We attribute this difference to our study’s focus on relatively young birth cohorts with a comparably high baseline prevalence of HEC.

Importantly, we found substantial variation by ethnic background. The Finnish sample, marked by higher baseline consumption, exhibited a substantively stronger reaction, showing a 17.7 % increase, while the Middle Eastern sample remained unaffected by the policy change. In the case of the Swedish sample, the increase was similar to that of the total population. One of the most challenging aspects of DID estimation is evaluating the assumption of parallel trends. Following current recommendations,^26^^,^^27^ we reported a number of statistical tests, graphical illustrations, and additional analyses that supported the credibility of our results. A cautionary note should nonetheless be included when interpreting the findings for the total population and the Swedish sample since the parallel trend did not hold when assessed over a pre-intervention period twice as long. Accordingly, the reminder of the discussion will focus primarily on the results for the Finnish and, to some extent, the Middle Eastern sample.

Among the individuals of Finnish origin, the impact of the reform was driven primarily by men and the youngest birth cohort. It can be noted that we also observed the similar patterns in the Swedish sample, albeit at lower levels. The findings are consistent with both prior research that has indicated sex and birth cohort differences in risk consumption^20^^,^^29^^‑^^32^ and the notion of risky consumers being more susceptible to changes in alcohol availability.^14^ Regarding education level in the Finnish (and Swedish) sample, there was no evidence of a gradient in the probability of HEC. Instead, the effect of the reform was most pronounced among those with upper secondary education. These findings contrast somewhat with previous studies, suggesting disproportionate health consequences of alcohol consumption among the lowest-educated group.^20^ There are a few possible explanations. For example, it could be due to our focus on relatively young cohorts, who might not yet have reached the educational potential. Additionally, lower-educated groups might be more likely than higher-educated groups to obtain alcoholic beverages from sources other than Systembolaget, such as smuggled and home-distilled alcohol.^33^ Therefore, extending Systembolaget’s sales hours would not matter as much for their consumption. Finally, concerning generation, we observed a statistically significant effect of the reform among second-generation migrants but not among first-generation migrants. This difference might be partly attributed to the composition of the group. In the 1970s, there was a substantial influx of Finnish migrants who had comparably lower education, worse health, and came to work in the industry.^34^ A large portion of individuals in our Finnish sample likely descend from these migrants, which could explain both their higher baseline level of HEC and why they might respond more strongly to the reform.

No measurable impact of the reform was found among individuals of Middle Eastern origin, which is to be expected since their level of alcohol consumption is known to be much lower. Even so, the large effect estimate (corresponding to a 21.4% decrease) warrants some discussion. Here it can be highlighted that the Middle Eastern sample was relatively small. In combination with the low prevalence of HEC, even small fluctuations across the time intervals can yield estimates that manifest as large—but statistically nonsignificant—effect sizes. An interesting exception was found for the group with university education, which merits further exploration in future studies.

### Strengths and limitations

Key strengths of this study include the use of a quasi-experimental DID design with multiple time periods (10 intervals, not just one before and one after the reform). Another strength is the massive sample size. The utilization of total population individual-level register data (*n* > 1 700 000) not only allowed for focusing on a relatively low-prevalent outcome such as HEC, it also enabled the examination of heterogeneity in responses to the reform by zooming in on smaller subgroups of the addressed population.

Quasi-experimental impact evaluations based on individual-level longitudinal register data have inherent limitations and our study is no exception. The prevalence of HEC was very low in some subgroups; a large sample size is thus not a guarantee for sufficient statistical power. Accordingly, it would perhaps have been better to use an outcome that captured a wider range of alcohol-related diagnoses. However, given that all studies include trade-offs, we preferred considering lag time from event (eg, many alcohol-related outcomes take time to manifest). Another potential limitation refers to whether and to what extent our outcome reflects heterogenous responses in itself, since it includes everything from accidents to suicide attempts. Regrettably, we were not able to examine the ICD codes at a more granular level due to data limitations. The most comparable Swedish statistics, derived for the year 2021, nonetheless show that accidents made up an exceedingly large proportion of HEC in our studied age group, followed by intentional self-harm, among men and women alike. Both specific types of outcomes have previous been shown to be sensitive to changes in alcohol policy, although these findings are not consistent in the literature.^35^^‑^^39^

### Implications for policy

Our results show that the Swedish Saturday opening reform primarily increased the risk of HEC in groups known to have more harmful patterns of alcohol consumption. This might indicate that increased alcohol availability adds to the disease burden attributable to alcohol among groups that are already vulnerable to its effects. While situated in the Swedish context, our results can inform ongoing discussions on changes in alcohol policy also in other country settings.

## Supplementary Material

Web_Material_kwae208

## Data Availability

The data that support the findings of this study are available from Statistics Sweden and the National Board of Health and Welfare but restrictions apply to the availability of these data, which were used under license for the current study, and so are not publicly available. Data are however available from the authors upon reasonable request and with permission of Statistics Sweden and the National Board of Health and Welfare.

## References

[1] Rehm J, Baliunas D, Borges GL, et al. The relation between different dimensions of alcohol consumption and burden of disease: an overview. *Addiction*. 2010;105(5):817-843. 10.1111/j.1360-0443.2010.02899.x 20331573 PMC3306013

[2] Burton R, Sheron N. No level of alcohol consumption improves health. *Lancet*. 2018;392(10152):987-988. 10.1016/S0140-6736(18)31571-X 30146328

[3] Babor TF, Casswell S, Graham K, et al. Alcohol: No Ordinary Commodity: Research and Public Policy. Wiley; 2022.

[4] Sherk A, Stockwell T, Chikritzhs T, et al. Alcohol consumption and the physical availability of take-away alcohol: systematic reviews and meta-analyses of the days and hours of sale and outlet density. *J Stud Alcohol Drugs*. 2018;79(1):58-67. 10.15288/jsad.2018.79.58 29227232

[5] Gruenewald PJ . Regulating availability: how access to alcohol affects drinking and problems in youth and adults. *Alcohol Res*. 2011;34(2):248-256. https://pmc.ncbi.nlm.nih.gov/articles/PMC3860569/PMC3860569 22330225

[6] Room R, Babor T, Rehm J. Alcohol and public health. *Lancet*. 2005;365(9458):519-530. 10.1016/S0140-6736(05)17870-2 15705462

[7] Anderson P, Chisholm D, Fuhr DC. Effectiveness and cost-effectiveness of policies and programmes to reduce the harm caused by alcohol. *Lancet*. 2009;373(9682):2234-2246. 10.1016/S0140-6736(09)60744-3 19560605

[8] Nelson JP, McNall AD. What happens to drinking when alcohol policy changes? A review of five natural experiments for alcohol taxes, prices, and availability. *Eur J Health Econ*. 2017;18(4):417-434. 10.1007/s10198-016-0795-0 27055901

[9] Giesbrecht N, Wettlaufer A, Cukier S, et al. Do alcohol pricing and availability policies have differential effects on sub-populations? A commentary. *Int J Alcohol Drug Res*. 2016;5(3):89-99. 10.7895/ijadr.v5i3.227

[10] Ekström KM, Hansson L. Systembolaget—alcohol monopoly and public health in Sweden. Global trends and success stories. Social marketing for. Public Health. 2009;171.19493353

[11] Grönqvist H, Niknami S. Alcohol availability and crime: lessons from liberalized weekend sales restrictions. *J Urban Econ*. 2014;81(2014):77-84. 10.1016/j.jue.2014.03.001

[12] Norström T, Skog O-J. Saturday opening of alcohol retail shops in Sweden: an impact analysis. *J Stud Alcohol*. 2003;64(3):393-401. 10.15288/jsa.2003.64.393 12817829

[13] Norström T, Skog OJ. Saturday opening of alcohol retail shops in Sweden: an experiment in 2 phases. *Addiction*. 2005;100(6):767-776. 10.1111/j.1360-0443.2005.01068.x 15918807

[14] Room R . Effects of alcohol controls: Nordic research traditions. *Drug Alcohol Rev*. 2004;23(1):43-53. 10.1080/095952304100001645547 14965886

[15] Fitzgerald N, Angus K, Emslie C, et al. Gender differences in the impact of population-level alcohol policy interventions: evidence synthesis of systematic reviews. *Addiction*. 2016;111(10):1735-1747. 10.1111/add.13452 27177685

[16] WHO . Global status report on alcohol and health 2018. 2018.

[17] Safiri S, Nejadghaderi SA, Noori M, et al. Burden of diseases and injuries attributable to alcohol consumption in the Middle East and North Africa region, 1990-2019. *Sci Rep*. 2022;12(1):19301. 10.1038/s41598-022-22901-x 36369336 PMC9652338

[18] Hjern A, Allebeck P. Alcohol-related disorders in first-and second-generation immigrants in Sweden: a national cohort study. *Addiction*. 2004;99(2):229-236. 10.1046/j.1360-0443.2003.00562.x 14756715

[19] WHO . *Status report on alcohol consumption, harm and policy responses in 30 European countries 2019*. 2019.

[20] Norström T, Landberg J. The link between per capita alcohol consumption and alcohol-related harm in educational groups. *Drug Alcohol Rev*. 2020;39(6):656-663. 10.1111/dar.13114 32654401

[21] Cook WK, Li X, Sundquist K, et al. Drinking cultures and socioeconomic risk factors for alcohol and drug use disorders among first-and second-generation immigrants: a longitudinal analysis of Swedish population data. *Drug Alcohol Depend*. 2021;226(2021):108804. 10.1016/j.drugalcdep.2021.108804 34216865 PMC8355220

[22] Wing C, Simon K, Bello-Gomez RA. Designing difference in difference studies: best practices for public health policy research. *Annu Rev Public Health*. 2018;39(1):453-469. 10.1146/annurev-publhealth-040617-013507 29328877

[23] StataCorp . *Didregress*. 2021.

[24] Jakobsen K, Jakobsen K, Kleven H, et al. Wealth taxation and wealth accumulation: theory and evidence from Denmark. *Q J Econ*. 2020;135(1):329-388. 10.1093/qje/qjz032

[25] Dobkin C, Finkelstein A, Kluender R, et al. The economic consequences of hospital admissions. *Am Econ Rev*. 2018;108(2):308-352. 10.1257/aer.20161038 30091560

[26] Freyaldenhoven S, Hansen C, Shapiro JM. Pre-event trends in the panel event-study design. *Am Econ Rev*. 2019;109(9):3307-3338. 10.1257/aer.20180609

[27] Roth J . Pretest with caution: event-study estimates after testing for parallel trends. *Am Econ Rev: Insights*. 2022;4(3):305-322.

[28] Avdic D, von Hinke S. Extending alcohol retailers’ opening hours: evidence from Sweden. *Eur Econ Rev*. 2021;138(2021):103830. 10.1016/j.euroecorev.2021.103830

[29] Slade T, Chapman C, Swift W, et al. Birth cohort trends in the global epidemiology of alcohol use and alcohol-related harms in men and women: systematic review and metaregression. *BMJ Open*. 2016;6(10):e011827. 10.1136/bmjopen-2016-011827 PMC5093369 27797998

[30] Leão TS, Johansson L-M, Sundquist K. Hospitalization due to alcohol and drug abuse in first-and second-generation immigrants: a follow-up study in Sweden. *Subst Use Misuse*. 2006;41(3):283-296. 10.1080/10826080500409100 16467006

[31] Iranpour A, Nakhaee N. A review of alcohol-related harms: a recent update. *Addict Health*. 2019;11(2):129-137. 10.22122/ahj.v11i2.225 31321010 PMC6633071

[32] Östergren O, Korhonen K, Gustafsson N-K, et al. Home and away: mortality among Finnish-born migrants in Sweden compared to native swedes and Finns residing in Finland. *Eur J Public Health*. 2021;31(2):321-325. 10.1093/eurpub/ckaa192 33230544 PMC8071591

[33] Norström T, Landberg J, Trolldal B. Drinking and acquisition of unrecorded alcohol across educational groups in Sweden. *Drug Alcohol Rev*. 2022;41(1):167-170. 10.1111/dar.13304 33960057

[34] Silventoinen K, Hammar N, Hedlund E, et al. Selective international migration by social position, health behaviour and personality. *Eur J Public Health*. 2007;18(2):150-155. 10.1093/eurpub/ckm052 17569701

[35] Trolldal B . An investigation of the effect of privatization of retail sales of alcohol on consumption and traffic accidents in Alberta. *Addiction*. 2005;100(5):662-671. 10.1111/j.1360-0443.2005.01049.x 15847624

[36] Nelson JP, McNall AD. Alcohol prices, taxes, and alcohol-related harms: a critical review of natural experiments in alcohol policy for nine countries. *Health Policy*. 2016;120(3):264-272. 10.1016/j.healthpol.2016.01.018 26861971

[37] Sanchez-Ramirez DC, Voaklander D. The impact of policies regulating alcohol trading hours and days on specific alcohol-related harms: a systematic review. *Inj Prev*. 2018;24(1):94-100. 10.1136/injuryprev-2016-042285 28647704

[38] Popova S, Giesbrecht N, Bekmuradov D, et al. Hours and days of sale and density of alcohol outlets: impacts on alcohol consumption and damage: a systematic review. *Alcohol Alcohol*. 2009;44(5):500-516. 10.1093/alcalc/agp054 19734159

[39] Mäkelä P, Herttua K, Martikainen P. The socioeconomic differences in alcohol-related harm and the effects of alcohol prices on them: a summary of evidence from Finland. *Alcohol Alcohol*. 2015;50(6):661-669. 10.1093/alcalc/agv068 26113490

