# Supplementary material for: Heterogeneous effects of increased availability of alcohol on hospitalization due to external causes: quasi-experimental evidence from the introduction of Saturday opening at Swedish alcohol retail stores
Source: Am J Epidemiol. 2024 Jul 16;194(6):1717–25. doi: 10.1093/aje/kwae208 (PMC12133268; doi:10.1093/aje/kwae208)
Supplement: Web_Material_kwae208 [file web_material_kwae208.docx]

**SUPPLEMENTARY MATERIAL**

**Title**

Heterogeneous effects of increased availability of alcohol on hospitalisation due to external causes. Quasi-experimental evidence from the introduction of Saturday opening at Swedish alcohol retail stores

**Authors**

Ylva B Almquist, Lars Brännström, Anders-Hjorth-Trolle, Mikael Rostila

**Table of contents**

Table S1. Distribution (n) of individuals within the ICD subchapters.

Table S2. Post-estimation tests. Based on the corresponding models from Table 4.

Table S3. Covariate imbalance between experiment and control area.

Table S4. Covariate imbalance between experiment and control area, after coarsened exact matching.*

Table S5. Effects of Saturday opening at Systembolaget on the probability of HEC across three-month intervals (November 1998 – April 2001). Results from fixed-effects DID models, weighed based on coarsened exact matching.*

Table S6. Effects of Saturday opening at Systembolaget on the probability of hospitalisation due to cancer* across three-month intervals (November 1998 – April 2001). Results from fixed-effects DID models.

Table S7. Effects of Saturday opening at Systembolaget on the probability of HEC across three-month intervals with an extended pre-intervention period* (August 1997 – April 2001). Results from fixed-effects DID models.

Table S8. Effects of Saturday opening at Systembolaget on the probability of HEC across three-month intervals. Pooled estimates* from the 1st phase (November 1998 – April 2001) and the 2nd phase of implementation (April 2000 – September 2002). Results from fixed-effects DID models.

Figure S1. Trend plots. Based on the corresponding models from Table 4. HEC=Hospitalisation due to external causes.

Figure S2. Granger plots of effects (with 95% confidence intervals). Based on the corresponding models from Table 4.

| Table S1. Distribution (n) of individuals within the ICD subchapters. | | | | | | | | | | | |
| --- | --- | --- | --- | --- | --- | --- | --- | --- | --- | --- | --- |
|  | **ICD codes** | **Nov98 – Jan 99** | **Feb 99 – Apr 99** | **May 99 – Jul 99** | **Aug 99 – Oct 99** | **Nov 99 – Jan 00** | **Feb 00 – Apr 00** | **May 00 – Jul 00** | **Aug 00 – Oct 00** | **Nov 00 – Jan 01** | **Feb 01 – Apr 01** |
| Total* | V01-Y98 | 3,649 | 3,807 | 4,333 | 4,005 | 3,778 | 3,789 | 3,916 | 3,910 | 3,849 | 3,759 |
| Self-harm | X60-X84 | 436 | 463 | 548 | 499 | 493 | 497 | 498 | 526 | 533 | 530 |
| Assault | X85-Y09 | 2 | 0 | 0 | 1 | 0 | 1 | 0 | 0 | 0 | 3 |
| Event of undetermined intent | Y10-Y34 | 44 | 66 | 60 | 43 | 58 | 50 | 65 | 47 | 57 | 44 |
| Legal intervention and op. of war | Y35-Y36 | 1 | 0 | 5 | 1 | 2 | 5 | 0 | 3 | 2 | 0 |
| Remaining ICD codes  Accidents  Compl. of medical and surgical care  Sequelae of external causes  Supplementary factors | V01-X59  Y40-Y84  Y85-Y89  Y90-Y98 | 3,176 | 3,304 | 3,743 | 3,490 | 3,250 | 3,184 | 3,372 | 3,358 | 3,286 | 3,204 |
| ICD=International Classification of Diseases.  * Any individual can appear in multiple subchapters. Therefore, sum of all individuals can exceed the Total. | | | | | | | | | | | |

| Table S2. Post-estimation tests. Based on the corresponding models from Table 4. | | |
| --- | --- | --- |
|  | **p-value for**  **parallel trends**^3^ | **p-value for**  **Granger test**^4^ |
| **Total** (n=1,782,412) | 0.8668 | **0.0007** |
| *Stratified by sex* |  |  |
| Men | 0.4927 | **0.0011** |
| Women | 0.5378 | 0.1279 |
| *Stratified by birth cohort* |  |  |
| Born 1959-1965 | 0.1532 | **0.0002** |
| Born 1966-1972 | 0.5636 | 0.9132 |
| Born 1973-1979 | 0.5124 | **0.0020** |
| *Stratified by educational level* |  |  |
| Compulsory education | 0.3997 | 0.2051 |
| Upper sec. education | 0.8526 | **0.0125** |
| University education | 0.2642 | 0.1444 |
| Missing education | 0.7820 | 0.5562 |
| *Stratified by generation* |  |  |
| First generation | 0.6531 | 0.2348 |
| Second generation | 0.6636 | **0.0120** |
| **Sweden** (n=1,189,718) | 0.4765 | 0.0984 |
| *Stratified by sex* |  |  |
| Men | 0.5968 | 0.2144 |
| Women | 0.6395 | 0.2242 |
| *Stratified by birth cohort* |  |  |
| Born 1959-1965 | **0.0239** | **0.0072** |
| Born 1966-1972 | 0.9234 | 0.8430 |
| Born 1973-1979 | 0.2744 | **0.0303** |
| *Stratified by educational level* |  |  |
| Compulsory education | 0.1764 | 0.7228 |
| Upper sec. education | 0.6616 | 0.0968 |
| University education | 0.5315 | 0.2781 |
| Missing education | 0.7532 | 0.0945 |
| **Finland** (n=113,038) | 0.8978 | 0.0939 |
| *Stratified by sex* |  |  |
| Men | 0.2401 | 0.2315 |
| Women | 0.2245 | 0.1399 |
| *Stratified by birth cohort* |  |  |
| Born 1959-1965 | 0.7853 | **0.0240** |
| Born 1966-1972 | 0.8061 | 0.6105 |
| Born 1973-1979 | 0.7108 | 0.3288 |
| *Stratified by educational level* |  |  |
| Compulsory education | 0.4253 | 0.6164 |
| Upper sec. education | 0.9145 | 0.2300 |
| University education | 0.2991 | 0.1930 |
| Missing education | 0.7555 | 0.6190 |
| *Stratified by generation* |  |  |
| First generation | 0.0838 | 0.4595 |
| Second generation | 0.3547 | **0.0440** |
| **Middle East** (n=45,848) | 0.7993 | 0.2061 |
| *Stratified by sex* |  |  |
| Men | 0.9224 | 0.1845 |
| Women | 0.6308 | 0.7720 |
| *Stratified by birth cohort* |  |  |
| Born 1959-1965 | **0.0126** | 0.0883 |
| Born 1966-1972 | 0.0539 | 0.1669 |
| Born 1973-1979 | 0.6971 | 0.4582 |
| *Stratified by educational level* |  |  |
| Compulsory education | 0.7625 | 0.5316 |
| Upper sec. education | 0.7588 | 0.6489 |
| University education | 0.5171 | 0.4627 |
| Missing education | 0.6674 | 0.1931 |
| *Stratified by generation* |  |  |
| First generation | 0.7456 | 0.2218 |
| Second generation | 0.3275 | 0.5560 |
| Estimates in bold signifies p<0.05.  ^1^ Null hypothesis (H0): Linear trends are parallel.  ^2^ Null hypothesis (H0): No effect in anticipation of treatment. | | |

| Table S3. Covariate imbalance between experiment and control area. | | | | |
| --- | --- | --- | --- | --- |
|  | **Total^1^**  (n=1,782,412) | **Sweden^2^**  (n=1,189,718) | **Finland^3^**  (n=113,038) | **Middle East^4^**  (n=45,848) |
| **Sex** |  |  |  |  |
| Men | 0.00979 | 0.00951 | 0.0266 | 0.01143 |
| Women | 0.00979 | 0.00951 | 0.0266 | 0.01143 |
| **Birth cohort** |  |  |  |  |
| Born 1959-1965 | 0.00345 | 0.00416 | 0.00402 | 0.00432 |
| Born 1966-1972 | 0.00506 | 0.00593 | 0.00298 | 0.00650 |
| Born 1973-1979 | 0.00161 | 0.00177 | 0.00700 | 0.00217 |
| **Educational level** |  |  |  |  |
| Compulsory education | 0.00984 | 0.01427 | 0.01979 | 0.01801 |
| Upper sec. education | 0.06012 | 0.05833 | 0.05843 | 0.02812 |
| University education | 0.06575 | 0.07276 | 0.07328 | 0.04528 |
| Missing education | 0.00421 | 0.00016 | 0.00495 | 0.00085 |
| **Generation** |  |  |  |  |
| First generation | 0.03657 | - | 0.02854 | 0.01538 |
| Second generation | 0.01348 | - | 0.02854 | 0.01538 |
| ^1^ The whole population regardless of ethnic background.  ^2^ Swedish-born with Swedish-born parents.  ^3^ Finnish-born or Swedish-born with a Finnish-born parent.  ^4^ Middle Eastern-born or Swedish-born with a Middle Eastern-born parent. | | | | |

| Table S4. Covariate imbalance between experiment and control area, after coarsened exact matching.* | | | | |
| --- | --- | --- | --- | --- |
|  | **Total^1^**  (n=1,782,412) | **Sweden^2^**  (n=1,189,718) | **Finland^3^**  (n=113,038) | **Middle East^4^**  (n=45,848) |
| **Sex** |  |  |  |  |
| Men | 9.1e-13 | 4.6e-13 | 4.7e-14 | 1.9e-14 |
| Women | 9.1e-13 | 4.6e-13 | 4.7e-14 | 1.9e-14 |
| **Birth cohort** |  |  |  |  |
| Born 1959-1965 | 9.1e-13 | 5.6e-13 | 2.5e-14 | 9.3e-15 |
| Born 1966-1972 | 1.1e-12 | 3.0e-13 | 5.8e-14 | 1.9e-14 |
| Born 1973-1979 | 9.7e-13 | 2.1e-13 | 3.4e-14 | 1.5e-14 |
| **Educational level** |  |  |  |  |
| Compulsory education | 2.9e-13 | 4.7e-13 | 2.0e-14 | 1.6e-14 |
| Upper sec. education | 8.6e-13 | 2.4e-13 | 2.7e-14 | 1.8e-14 |
| University education | 7.3e-13 | 1.4e-13 | 1.3e-14 | 6.5e-15 |
| Missing education | 2.1e-14 | 6.1e-15 | 1.4e-15 | 6.9e-15 |
| **Generation** |  |  |  |  |
| First generation | 3.8e-13 | - | 4.7e-14 | 1.6e-15 |
| Second generation | 5.4e-13 | - | 4.7e-14 | 1.6e-15 |
| *Coarsened exact matching performed within Total/Sweden/Finland/Middle East. Matching based on sex, birth cohort, educational level, and generation (where appropriate).  ^1^ The whole population regardless of ethnic background.  ^2^ Swedish-born with Swedish-born parents.  ^3^ Finnish-born or Swedish-born with a Finnish-born parent.  ^4^ Middle Eastern-born or Swedish-born with a Middle Eastern-born parent. | | | | |

| Table S5. Effects of Saturday opening at Systembolaget on the probability of HEC across three-month intervals (November 1998 – April 2001). Results from fixed-effects DID models, weighed based on coarsened exact matching.* | | | | | |
| --- | --- | --- | --- | --- | --- |
|  | **Estimate^1^** | **Robust**  **std. err.** | **p-value** | **p-value**  **for parallel trends**^2^ | **p-value**  **for Granger**  **test**^3^ |
| **Total** (n=1,782,412) | **0.00016** | 0.00005 | **0.001** | 0.9110 | **0.0005** |
| **Sweden** (n=1,189,718) | **0.00020** | 0.00005 | **<0.001** | 0.6679 | 0.1087 |
| **Finland** (n=113,038) | **0.00048** | 0.00020 | **0.017** | 0.9513 | 0.0542 |
| **Middle East** (n=45,848) | -0.00056 | 0.00029 | 0.055 | 0.8640 | 0.2336 |
| *Coarsened exact matching performed within Total/Sweden/Finland/Middle East. Matching based on sex, birth cohort, educational level, and generation (where appropriate).  HEC=Hospitalisation due to external causes. DID=Difference-in-differences.  Estimates in bold signifies p<0.05.  ^1^ Estimates are absolute differences in probabilities (risk differences).  ^2^ Null hypothesis (H0): Linear trends are parallel.  ^3^ Null hypothesis (H0): No effect in anticipation of treatment. | | | | | |

| Table S6. Effects of Saturday opening at Systembolaget on the probability of hospitalisation due to cancer* across three-month intervals (November 1998 – April 2001). Results from fixed-effects DID models. | | | | |
| --- | --- | --- | --- | --- |
|  | **Prevalence** | **Estimate^1^** | **Robust**  **std. err.** | **p-value** |
| **Total** (n=1,782,412) | 0.93 | 0.00004 | 0.00002 | 0.065 |
| **Sweden** (n=1,189,718) | 0.87 | 0.00005 | 0.00003 | 0.076 |
| **Finland** (n=113,038) | 0.98 | 0.00000 | 0.00009 | 0.993 |
| **Middle East** (n=45,848) | 1.14 | 0.00017 | 0.00017 | 0.306 |
| * Based on Chapter II in ICD10 (Neoplasms).  DID=Difference-in-differences.  Estimates in bold signifies p<0.05.  ^1^ Estimates are absolute differences in probabilities (risk differences). | | | | |

| Table S7. Effects of Saturday opening at Systembolaget on the probability of HEC across three-month intervals with an extended pre-intervention period* (August 1997 – April 2001). Results from fixed-effects DID models. | | | | | |
| --- | --- | --- | --- | --- | --- |
|  | **Estimate^1^** | **Robust**  **std. err.** | **p-value** | **p-value**  **for parallel trends**^2^ | **p-value**  **for Granger**  **test**^3^ |
| **Total** (n=1,782,412) | 0.00031 | 0.00004 | <0.001 | **<0.001** | **<0.001** |
| **Sweden** (n=1,189,718) | 0.00034 | 0.00005 | <0.001 | **<0.001** | **<0.001** |
| **Finland** (n=113,038) | 0.00034 | 0.00018 | 0.052 | 0.5727 | 0.1069 |
| **Middle East** (n=45,848) | -0.00028 | 0.00025 | 0.261 | 0.4185 | 0.0987 |
| * The pre-intervention period is divided into 10 time intervals: August 1997 – October 1997; November 1997 – January 1998; February 1998 – April 1998; May 1998 – July 1998; August 1998 – October 1998; November 1998 – January 1999; February 1999 – April 1999; May 1999 – July 1999; August 1999 – October 1999; November 1999 – January 2000. The post-intervention period is the same as in the main analyses.  HEC=Hospitalisation due to external causes. DID=Difference-in-differences.  Estimates in bold signifies p<0.05.  ^1^ Estimates are absolute differences in probabilities (risk differences).  ^2^ Null hypothesis (H0): Linear trends are parallel  ^3^ Null hypothesis (H0): No effect in anticipation of treatment. | | | | | |

| Table S8. Effects of Saturday opening at Systembolaget on the probability of HEC across three-month intervals. Pooled estimates* from the 1^st^ phase (November 1998 – April 2001) and the 2^nd^ phase of implementation (April 2000 – September 2002). Results from fixed-effects DID models. | | | | | |
| --- | --- | --- | --- | --- | --- |
|  | **Estimate^1^** | **Robust**  **std. err.** | **p-value** | **p-value**  **for parallel trends**^2^ | **p-value**  **for Granger**  **test**^3^ |
| **Total** (n=1,782,412) | **0.00008** | 0.00003 | 0.009 | 0.360 | 0.114 |
| **Sweden** (n=1,189,718) | **0.00012** | 0.00004 | 0.002 | 0.249 | 0.600 |
| **Finland** (n=113,038) | 0.00017 | 0.00014 | 0.230 | 0.532 | 0.102 |
| **Middle East** (n=45,848) | **-0.00040** | 0.00020 | 0.045 | 0.745 | 0.151 |
| * The dataset for the first implementation phase corresponds to the one used in the main analyses. The dataset for the second implementation phase is set up in the same way with five time intervals across the pre-intervention period (April 2000 – June 2000; July 2000 – September 2000; October 2000 – December 2000; January 2001 – March 2001; April 2001 – June 2001) and five time intervals across the post-intervention period (July 2001 – September 2001; October 2001 – December 2001; January 2002 – March 2002; April 2002 – June 2002; July 2002 – September 2002). The two datasets have subsequently been appended (stacked), based on which the DID estimation is performed.  In practice, this means that the experiment area in the 1^st^ phase by necessity becomes the control area in the 2^nd^ phase (and vice versa). Accordingly, for the 2^nd^ phase, we compare the treated to the previously treated. Concrete illustrations of the three main problems that arise from this approach are presented in a recent publication by Baker and colleagues.^1^ First, groups treated at different times may also have different treatment effects, which causes problems with the OLS variance weights. Second, we could have dynamic treatment effects, so that the treatment does not only change the level of the dependent variable, but also its development over time, which causes serious comparison problems. Third, if we combine these two scenarios, one might even run the risk of estimating the sign of the effect wrongly. It should also be noted that each of these models assume that we have a group of individuals that never received the treatment, which we do not have in the pooled models. For a further discussion of these issues, see also ^2-4^.  HEC=Hospitalisation due to external causes. DID=Difference-in-differences.  Estimates in bold signifies p<0.05.  ^1^ Estimates are absolute differences in probabilities (risk differences).  ^2^ Null hypothesis (H0): Linear trends are parallel.  ^3^ Null hypothesis (H0): No effect in anticipation of treatment. | | | | | |

| A. Total (n=1,782,412) | B. Sweden (n=1,189,718) |
| --- | --- |
|  |  |
| **C. Finland** (n=113,038) | **D. Middle East** (n=45,848) |
| **** | **** |
| Figure S1. Trend plots. Based on the corresponding models from Table 4. HEC=Hospitalisation due to external causes. | |

| A. Total (n=1,782,412) | B. Sweden (n=1,189,718) |
| --- | --- |
| **** | **** |
| **C. Finland** (n=113,038) | **D. Middle East** (n=45,848) |
| **** | **** |
| Figure S2. Granger plots of effects (with 95% confidence intervals). Based on the corresponding models from Table 4. | |

**REFERENCES**

1. Baker AC, Larcker DF, Wang CC. How much should we trust staggered difference-in-differences estimates? *Journal of Financial Economics*. 2022;144(2):370-395.

2. Goodman-Bacon A. Difference-in-differences with variation in treatment timing. *Journal of Econometrics*. 2021;225(2):254-277.

3. De Chaisemartin C, d’Haultfoeuille X. Two-way fixed effects estimators with heterogeneous treatment effects. *American Economic Review*. 2020;110(9):2964-2996.

4. Sant’Anna PH, Zhao J. Doubly robust difference-in-differences estimators. *Journal of Econometrics*. 2020;219(1):101-122.
